# Pensions for Nurses

**Published:** 1919-10-25

**Authors:** 


### THE MATRONS' AND SISTERS' DEPARTMENT.

### PENSIONS FOR NURSES.

There is a growing feeling among those who know the facts that to put the nursing profession on a proper footing of independence it is essential to have a comprehensive scheme of pensions on a contributory basis. To a very large extent the ordinary incentives to thrift have been seen to break down. Quite apart from the distress occasioned by rapid demobilisation and by the risks of war work, there is a vast amount of distress among nurses. Sifted with care the cases fall into two categories, those who could have made provision for sickness and old age and those who could not by any stretch of economy have succeeded in doing so. Within the latter category fall hundreds of women whose days have been a perpetual struggle against poverty. We think our readers would have no difficulty in reckoning up dozens of nurses in their acquaintance who at the present moment are not in receipt of a living wage, if by living wage be meant one which enables them to secure a living in old age. The need for a comprehensive scheme of pensions for nurses unable to ensure their future is so obvious that it shouts. It is one of the planks in nursing reform that every institute, every institution, every society which depends on nurses and uses up the best years of their lives should take out for them an adequate policy in the Royal National Pension Fund for Nurses. There are several, we would there were more, hospitals which act on this principle. There are several County Nursing Associations which make their pension quasi-compulsory and highly advantageous to the nurses. But, alas! the proportion of governors and committees which concern themselves with the nurse's future after she leaves their service is small indeed. At most "they draw the attention" of their nurses to possibilities of saving and leave them to sink or swim.

From this large class of nurses on salaries too small to give them a chance of saving, springs that multitude of nurses driven to seek aid from charitable sources which constitutes a blot on the profession. Were this all it would still be bad, but at least the problem would be simple. It would be proclaimed in season and out of season that the distress among nurses is caused by the low rate of pay, and the public would, we believe, soon see to it that the pay should be raised.

But everyone knows that by no means all the nurses who fall into distress are in this category. There are far too many nurses who are in trouble because they lived up to a good income during their best earning years; or imposed upon themselves burdens they could not meet unless their work remained for many years at high-water mark; or who squandered their savings on manifestly foolish investments; or succumbed to the confidence trick and entrusted their money to a casual acquaintance. When these cases come to light the need for a little gentle compulsion in the matter of placing savings becomes evident. We are often reiterating that nurses are not children. But at the same time we have to admit that the semi-conventual existence that they lead in hospitals, nursing institutes, and district work, disqualifies them from being considered business women. Unquestionably, if salaries were raised to-morrow to the figure we should like to see, there would be nurses in dire distress before five years were out. For a very large proportion of nurses (as of other women) regard a rise in salary as so much more money to spend and lack the imagination which would bring before them the lean years of the future. Unless saving is made compulsory higher salaries will not succeed in stabilising the nurse's lot.

We know no system better than that which is practised in certain hospitals. At the moment when the probationer enters the service the hospital takes out a policy in the Royal National Pension Fund for Nurses on condition that the nurse does the same. Every step in her career is marked by an increase in the sum paid on her account and in the sum paid in by her. The policies become her own on leaving. But this is the weak point. Were the pension scheme truly comprehensive this contributory system would be continued under whatever service she next accepts. Every hospital, every nursing institute, or district association, every municipal service she enters would carry out the same principle, paying their share of the policy in proportion to the nurse's contribution, and making this co-operation in saving a condition of service. In this way right through life the nurse would be guided to make her future secure and the whole nursing service would be set on an independent and really self-supporting basis.

